# Off-Target Effect of Endogenous siRNA Derived from *RMRP* in Human Cells

**DOI:** 10.3390/ijms14059305

**Published:** 2013-04-29

**Authors:** Yoshiko Maida, Satoru Kyo, Timo Lassmann, Yoshihide Hayashizaki, Kenkichi Masutomi

**Affiliations:** 1 Division of Cancer Stem Cell, National Cancer Center Research Institute, 5-1-1 Tsukiji, Chuo-ku, Tokyo 104-0045, Japan; 2 Department of Obstetrics and Gynecology, Kanazawa University School of Medicine, 13-1 Takaramachi, Kanazawa, Ishikawa 920-8641, Japan; E-Mail: satoruky@med.kanazawa-u.ac.jp; 3 RIKEN Omics Science Center, RIKEN Yokohama Institute, 1-7-22 Suehiro-cho, Tsurumi-ku, Yokohama 230-0045, Japan; E-Mails: lassmann@gsc.riken.jp (T.L.); yosihide@gsc.riken.jp (Y.H.)

**Keywords:** endogenous siRNA, hTERT, off-targeting, RNA-dependent RNA polymerase

## Abstract

Endogenous siRNAs (endo-siRNAs) are key regulators of RNA silencing in plants and worms; however, the biogenesis and function of endogenous siRNAs in mammals remain largely unknown. We previously demonstrated that human telomerase reverse transcriptase produces a self-targeting endogenous siRNA from non-coding *RMRP* RNA via RNA-dependent RNA polymerase (RdRP) activity. Here, we investigated whether the endo-siRNA derived from *RMRP* targets other genes in addition to *RMRP*. Four algorithms for microRNA target prediction were used to identify possible targets of the endo-siRNA, and the phytanoyl-CoA hydroxylase-interacting protein-like gene (*PHYHIPL*) was identified as the most promising candidate. The 3′ UTR of *PHYHIPL* was found to contain three possible target sites with perfect seed pairing; deletion of each of these sites resulted in recovery of upstream luciferase expression. In addition, sequence-specific inhibition of the *RMRP*-derived endo-siRNA increased expression of *PHYHIPL* mRNA. The results described here suggest that the endo-siRNA uses silencing mechanisms that are similar to those used by microRNAs for gene silencing. To our knowledge, this study is the first confirmation of the off-target effect of human endogenous siRNA produced by RdRP activity.

## 1. Introduction

RNA silencing is a sequence-based gene regulatory system that occurs almost ubiquitously across multiple organisms. Small interfering RNAs (siRNAs) and microRNAs (miRNAs) are double-stranded small RNA species of ~22 nucleotides in length that play central roles in RNA silencing. The guide strand of siRNAs and miRNAs binds to complementary sequences on target RNAs and mediates their degradation and/or translational inhibition. Although siRNAs and miRNAs share many common structural and functional features, the biosynthetic pathways of these small RNA species are quite different [1]. miRNAs are endogenously transcribed from genomic DNA as characteristic stem-loop base pairings in single-stranded precursor RNAs. The stem-loop structures are successively processed by Drosha and Dicer to generate mature miRNA duplexes. Conversely, template sequences for double-stranded siRNA precursors are not present in genomic DNA. Double-stranded siRNAs are endogenously synthesized by RNA-dependent RNA polymerase (RdRP), which creates RNA strands that are complementary to single-stranded template RNAs [2]. The double-stranded RdRP products are then processed into functional siRNAs in either a Dicer-dependent or a Dicer-independent manner. RdRP is therefore thought to be indispensable for RNA silencing by endogenous siRNA (endo-siRNA). Plants and nematodes utilize multiple RdRPs for different modes of endo-siRNA generation [3]. In these organisms, RdRPs amplify RNA silencing signals by copying target sequences to generate endogenous secondary siRNAs. However, molecules that display RdRP-like activities had not yet been identified in mammals. Consequently, investigation of the endogenous “copy and cut” mechanism performed by RdRPs and endo-siRNAs had been neglected.

We recently reported that human telomerase reverse transcriptase (hTERT) works as an RdRP in human cells [4]. hTERT interacts with the RNA component of mitochondrial RNA processing endoribonuclease (*RMRP*), a noncoding RNA of 267 nucleotides in length, and exhibits RdRP activity *in vitro* and *in vivo* [4]. *RMRP* is processed into a double-stranded RNA with a hairpin structure by the RdRP activity of hTERT and then diced into endo-siRNA. The endo-siRNA derived from *RMRP* is 22 nucleotides in length, and both the sense and antisense strands of the *RMRP*-derived endo-siRNA can be detected by Northern blotting using probes covering nucleotides 21–40 of *RMRP* [4]. The sense strand of the endo-siRNA, which is thought to be the guide strand, is selectively loaded onto protein argonaute-2 (AGO2). The endo-siRNA derived from *RMRP* targets and regulates *RMRP* itself [4].

Synthetic siRNAs have been widely adopted as a technology for sequence-based gene silencing. In contrast to miRNAs, which only require target pairing at the seed region (nucleotides 2–7) [5], it was initially thought that siRNAs are only capable of suppressing target RNAs containing perfectly complementary sequences; thus, gene silencing by siRNA is highly specific. However, recent studies have revealed that siRNA-mediated silencing is less specific than was originally believed. Synthetic exogenous siRNAs can regulate multiple unintended (off-target) genes, as well as their intended (on-target) genes [6,7]. Off-target effect of exogenous siRNA suggested a hypothesis that endogenous siRNA may also regulate unintended genes. Here, we report that the endo-siRNA derived from *RMRP* guides off-target effect via a mechanism similar to that of miRNAs.

## 2. Results and Discussion

### 2.1. The Endo-siRNA Sequence Originates from Nucleotides 22–43 of *RMRP*

**Figure 1:**
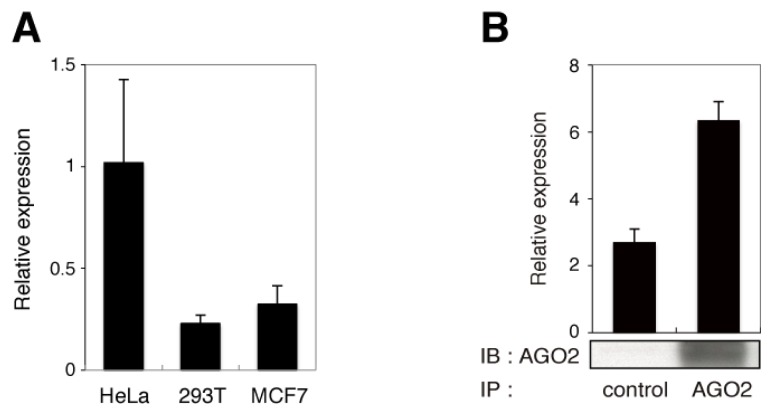
The endo-siRNA derived from *RMRP* originates from nucleotides 22–43 of *RMRP*. (**A**) Expression levels of the region of *RMRP* spanning nucleotides 22–43 in HeLa, 293T and MCF7 cells were analyzed by qRT-PCR. Data are represented as the mean ± SD for *n* = 3 independent experiments and are normalized to expression of the *RNU6B* gene. (**B**) Expression levels of the region of *RMRP* spanning nucleotides 22–43 in RNAs immunoprecipitated (IP) with an anti-AGO2 antibody from HeLa cells. Data are represented as the mean ± SD for *n* = 3 independent experiments. The results of immunoblotting (IB) with the anti-AGO2 antibody are also shown.

To explore novel target gene(s) of the endo-siRNA derived from *RMRP*, we determined the precise sequence of the endo-siRNA. Based on our previous study [4], the guide strand of the endo-siRNA was predicted to be 22 nucleotides in length and to overlap with nucleotides 21–40 of *RMRP*. Small RNAs that satisfied these criteria were searched from deep sequencing analysis of the THP-1 cell-line, and six candidate sequences were selected (Table 1). RNAs corresponding to nucleotides 22–43 (or 42) of *RMRP* were speculated to be the most likely candidates for two reasons. First, the calculated melting temperatures of the remaining sequences were too low to be detected by the Northern blotting method previously used to detect the endo-siRNA derived from *RMRP*, in which hybridization was performed at 37 °C with a DNA probe complementary to nucleotides 21–40 of *RMRP* [4]. Second, the endo-siRNA was detected with a probe complementary to nucleotides 22–33 of *RMRP*, but not detected with a probe complementary to nucleotides 44–55 (data not shown). To confirm the deep sequencing results, quantitative RT-PCR (qRT-PCR) analyses of the most likely candidate were performed using primers and probes specifically designed to detect nucleotides 22–43 of *RRMP*. Specific products were amplified from three different cell-lines (HeLa, 293T and MCF7), all of which have previously been shown to express the endo-siRNA derived from *RMRP* (Figure 1A). The expression levels were highest in the HeLa cell-line, which agrees with our previous Northern blotting data [4].

To investigate a physical interaction between the candidate RNA and Argonaute protein, AGO2 was immunoprecipitated from HeLa cells, and total RNA was then extracted from the precipitate and amplified by qRT-PCR using primers and probes specific to nucleotides 22–43 of *RMRP*. The candidate RNA was enriched in the RNAs immunoprecipitated with AGO2 (Figure 1B). Taken together, these data indicate that the endo-siRNA derived from *RMRP* originates from nucleotides 22–43 of *RMRP*.

### 2.2. The Endo-siRNA Derived from *RMRP* Mediates Gene Silencing

**Figure 2:**
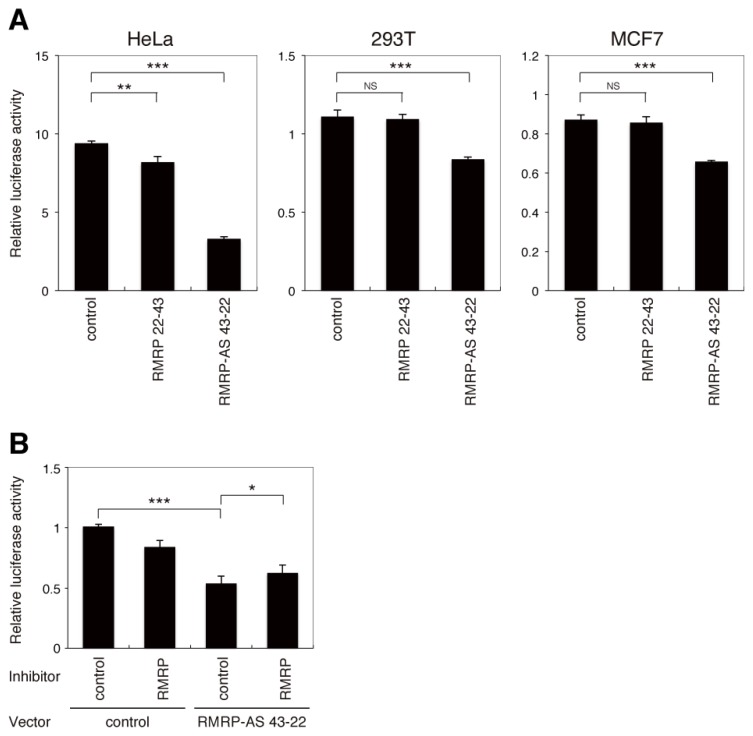
The endo-siRNA derived from *RMRP* exhibits gene silencing activity. (**A**) Cells were transfected with psiCHECK-2 vectors in which sequences identical (RMRP 22-43) or complementary (RMRP-AS 43-22) to the *RMRP*-derived endo-siRNA sequence were inserted into the 3′ UTR of the *Renilla* luciferase gene. Control cells were transfected with the native psiCHECK-2 vector. Luciferase activity was measured 48 h post-transfection. Data are represented as the mean ± SD of *n* = 3 independent experiments and are normalized to luciferase activity of the firefly luciferase gene in the psiCHECK-2 vector. (**B**) Luciferase activity in 293T cells transiently co-transfected with the indicated psiCHECK-2 vectors and a sequence-specific inhibitor of the *RMRP*-derived endo-siRNA (RMRP) or miScript Inhibitor Negative Control (control). Luciferase activity was measured 48 h post-transfection. Data are represented as the mean ± SD of *n* = 3 independent experiments and are normalized to luciferase activity of the firefly luciferase gene in the psiCHECK-2 vector. *p < 0.05; **p < 0.01; ***p < 0.001; NS, not significant; by two-tailed Student’s *t*-test.

To determine whether the endo-siRNA derived from nucleotides 22–43 of *RMRP* is functional, sequences identical to (RMRP 22–43) or complementary to (RMRP-AS 43–22) the endo-siRNA were inserted into the 3′ UTR of the *Renilla* luciferase gene in the psiCHECK-2 vector. The vectors with or without (control) inserted sequences were transfected into HeLa, 293T and MCF7 cells and luciferase activity was measured (Figure 2A). Insertion of RMRP-AS 43–22 specifically and significantly decreased expression of *Renilla* luciferase in all three cell types. This suppression of the luciferase gene was attenuated when a sequence-specific inhibitor of the *RMRP*-derived endo-siRNA was simultaneously transfected into cells (Figure 2B). These data indicate that the endo-siRNA derived from nucleotides 22–43 of *RMRP* mediates sequence-specific gene silencing.

### 2.3. Overall Sequence Analogy Does Not Accurately Predict Unintended Targets of the Endo-siRNA Derived from *RMRP*

**Figure 3:**
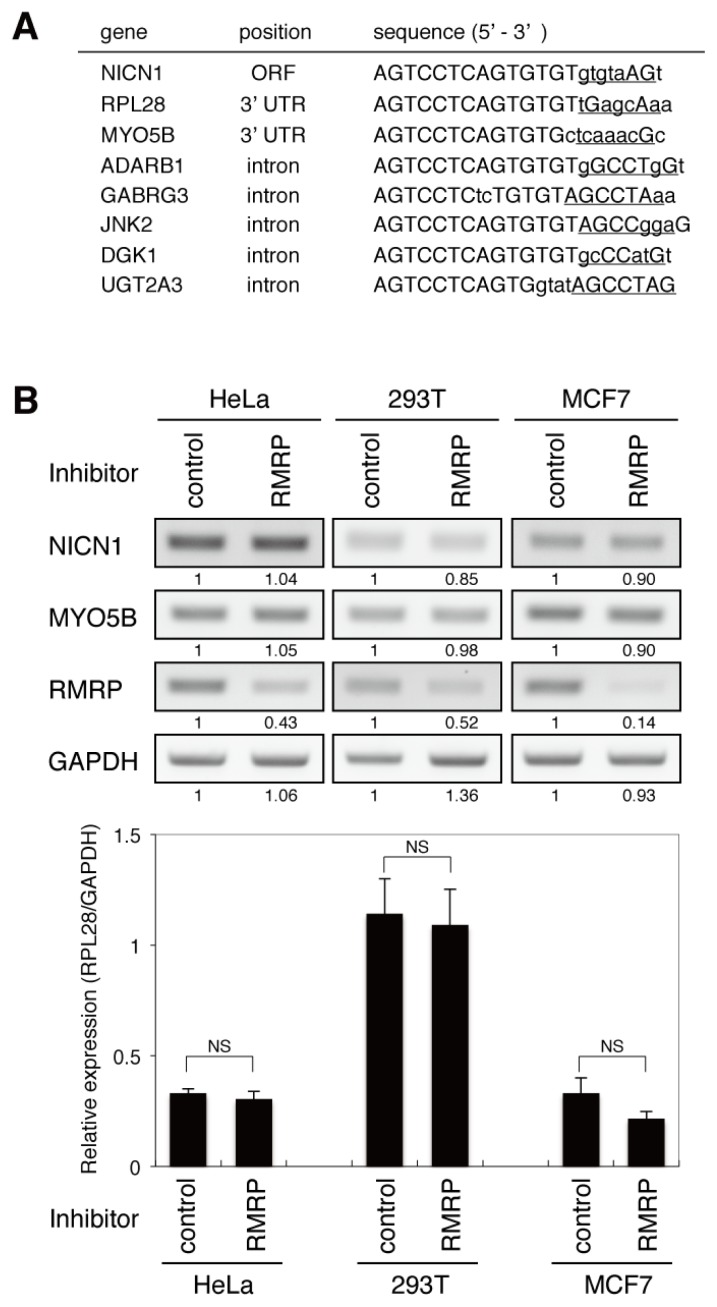
Sequence analogy is not predictive of *RMRP*-derived endo-siRNA target genes. (**A**) List of candidate targets of the endo-siRNA derived from *RMRP*. The candidates were selected based on their analogy to the sequence complementary to that of the endo-siRNA. Upper-case and lower-case letters indicate matched and mismatched pairing to the endo-siRNA sequence, respectively. Sequences corresponding to the seed region of the endo-siRNA are underlined. (**B**) RT-PCR (upper panels) and qRT-PCR (lower panel) analyses of the three candidate genes containing matched sequences located within an ORF or UTR. Total RNA was prepared from cells transfected with a sequence-specific inhibitor of the *RMRP*-derived endo-siRNA (RMRP) or miScript Inhibitor Negative Control (control). In the upper panels, numbers indicated under each panel represent relative signal intensity measured by densitometry. In the lower panel, data are represented as the mean ± SD of *n* = 3 independent experiments and are normalized to expression of the *GAPDH* gene. NS, not significant; by two-tailed Student’s *t*-test.

On-target gene regulation by siRNAs typically involves recognition of perfectly complementary sequences. Therefore, to identify candidate targets of the endo-siRNA derived from *RMRP*, BLAST searches were performed using the sequence complementary to the endo-siRNA. Although *RMRP* was the only gene that contained a perfectly matched sequence, a number of other genes included partially complementary sequences (Figure 3A). Notably, complementarity of each of the candidate sequences was higher at the 5′ end than at the 3′ end. A previous study suggested that exogenous siRNAs may not target intronic sequences [8]; therefore, we focused on the three candidate sequences (*NICN1*, *MYO5B* and *RPL28*) that were located within an ORF or UTR. To evaluate regulation of these genes by the endo-siRNA derived from *RMRP*, a sequence-specific inhibitor of the endo-siRNA was transfected into HeLa, 293T and MCF7 cells and the expression levels of *NICN1*, *MYO5B* and *RPL28* were analyzed by RT-PCR or qRT-PCR. Whereas *RMRP* expression was suppressed by the endo-siRNA inhibitor as expected, expression levels of *NICN1*, *MYO5B* and *RPL28* were not affected (Figure 3B), suggesting that expression of these candidate off-target genes is not regulated by the endo-siRNA derived from *RMRP*.

A previous study that analyzed the pattern of sequence alignment between six siRNAs and their off-target transcripts found that the regions of complementarity between the guide strand of the siRNA and the target transcript differs between mRNA regions [9]. The authors of this study reported that the longest contiguous region within each sequence alignment had an average length of eight nucleotides in both UTRs and coding sequences. Sequence matches in the 5′ UTR and coding sequence of off-target transcripts were biased towards the central region of the siRNA, whereas 3′ UTR matches showed bias towards the 5′ end of the siRNA guide strand [9]. A previous study that compared experimentally validated off-target transcripts with *in silico* predicted off-target transcripts, the latter of which were identified as gene sequences displaying ≥79% identity to either the sense or antisense strands of the specific siRNAs, reported unacceptably high false positive and false negative rates of the predicted off-target sequences [10]. The authors of this study concluded that overall sequence identity is a poor predictor of off-target genes [10]. Here, candidate targets of the endo-siRNA derived from *RMRP* were selected based on overall sequence analogy identified by a BLAST search; however, off-targeting of these candidates by the endo-siRNA was not verified experimentally. The endo-siRNA target sequence identified in *NICN1* was positioned within the ORF of the gene. The target sequence contained 14 contiguous nucleotides that were complementary to the central region of the *RMRP*-derived endo-siRNA; however, 73% (16/22) of sequence complementarity would explain the false positive prediction of this candidate. In contrast, the target sequences in *RPL28* and *MYO5B* were located within the 3′ UTRs of the genes, and the lower degree of complementarity to the 5′ end of the endo-siRNA would prevent off-targeting of these genes.

### 2.4. The Endo-siRNA Derived from RMRP Mediates Off-Targeting by a Mechanism Similar to That of miRNAs

Off-target effects of exogenous siRNAs fall into three major categories [6]: siRNA-induced sequence-dependent regulation of unintended genes through partial complementarity to 3′ UTRs (miRNA-like off-target effects), an inflammatory response triggered by siRNAs and/or their delivery vehicles, and widespread effects on miRNA processing and functions via saturation of the endogenous RNA interference machinery. We hypothesized that the miRNA-like mechanism is most likely to be applicable to off-targeting by endo-siRNAs, because these species are generated intracellularly and stably co-exist with miRNAs. Emerging evidence about this type of off-targeting has led to the consensus opinion that a sequence match at the seed region of a siRNA is indispensable for target recognition [6,9–13]. In addition, some reports have suggested that 3′ pairing at nucleotides 12–17 is also an important determinant of target recognition [10,12].

**Figure 4:**
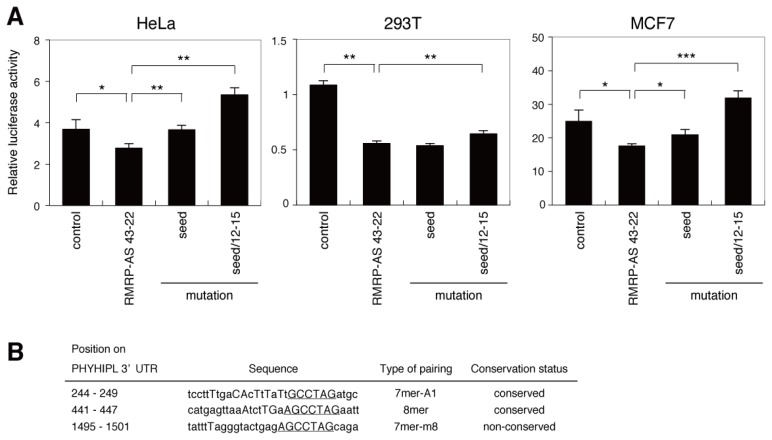

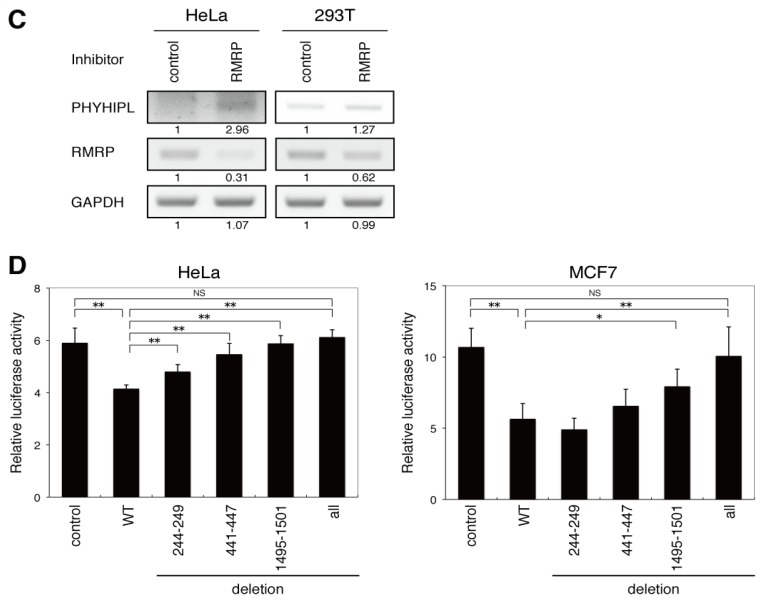
miRNA-like off-target effect of the endo-siRNA derived from *RMRP*. (**A**) Sequences complementary to the *RMRP*-derived endo-siRNA, with or without mutations in the seed region alone (seed) or the seed region and nucleotides 12–15 (seed/12–15), were inserted into the psiCHECK-2 vector. HeLa, 293T and MCF7 cells were transfected with the vectors and luciferase activity was measured 48 h post-transfection. Data are represented as the mean ± SD for *n* = 3 independent experiments and are normalized to luciferase activity of the firefly luciferase gene in the psiCHECK-2 vector. (**B**) Characteristics of the seed-matched sequences located within the 3′ UTR of *PHYHIPL*. Upper-case and lower-case letters indicate matched and mismatched pairing to the endo-siRNA derived from *RMRP*, respectively. Sequences corresponding to the seed region of the endo-siRNA are underlined. (**C**) RT-PCR analyses of *PHYHIPL* and *RMRP* mRNAs. Total RNAs were prepared from HeLa and 293T cells transfected with a sequence-specific inhibitor of the *RMRP*-derived endo-siRNA (RMRP) or miScript Inhibitor Negative Control (control). Numbers indicated under each panel represent relative signal intensity measured by densitometry. (**D**) HeLa and MCF7 cells were transfected with psiCHECK-2 vectors containing the wild-type (WT) or various deletion mutants of the *PHYHIPL* 3′ UTR. The numbers indicate the regions deleted; “all” indicates that all three regions were deleted. Luciferase activity was measured 48 h post-transfection. Data are represented as the mean ± SD of *n* = 3 independent experiments and are normalized to luciferase activity of the firefly luciferase gene in the psiCHECK-2 vector. *p < 0.05; **p < 0.01; ***p < 0.001; NS, not significant; by two-tailed Student’s *t*-test.

A luciferase assay was performed to examine whether sequence matches at the seed region and nucleotides 12–15 are important for gene suppression by the endo-siRNA derived from *RMRP*. Wild-type (RMRP-AS 43-22) or mutated (seed, seed/12–15) sequences complementary to the endo-siRNA were inserted into the 3′ UTR of the luciferase gene in the psiCHECK-2 vector. Compared with control cells, and consistent with the data shown in Figure 2A, luciferase activity was significantly lower in HeLa, 293T and MCF7 cells transfected with the vector containing wild-type RMRP-AS 43-22 (Figure 4A). The statistically significant suppression of the luciferase gene by RMRP-AS 43-22 was reproducible in these cells. Mutation of the seed region significantly attenuated the gene silencing effect of RMRP-AS 43-22 in HeLa and MCF7 cells, and double mutation of both the seed region and nucleotides 12–15 attenuated the silencing effect further in all three cell types (Figure 4A). The seed sequences of three human miRNAs (hsa-miR-645, hsa-miR-603 and hsa-miR-1224-5p) were identified within the endo-siRNA derived from *RMRP* (Supplementary Figure S1A). To investigate the possibility that the observed suppression of the luciferase gene by RMRP-AS 43-22 was caused by the seed-overlapping miRNAs rather than the *RMRP*-derived endo-siRNA, expression levels of these miRNAs in HeLa, 293T and MCF7 cells were analyzed by qRT-PCR (Supplementary Figure S1B). Although hsa-miR-1224-5p was detected in all cell types, hsa-miR-645 and hsa-miR-603 were not. The seed sequence of hsa-miR-1224-5p is located at the 3′ end of the endo-siRNA derived from *RMRP*. Although expression of this miRNA was detected, this region is not required for gene suppression by the endo-siRNA, because mutation of both the seed region and nucleotides 12–15 completely abolished the gene silencing effect of RMRP-AS 43-22 (Figure 4A). These results indicate that both the seed region and the 3′ pairing region are important for target recognition at 3′ UTRs by the endo-siRNA derived from *RMRP*.

Based on the results described above, potential novel targets of the *RMRP*-derived endo-siRNA were identified by focusing on 3′ UTR sequences complementary to both the seed region and the 3′ pairing region of the endo-siRNA. No genes containing both regions in the expected positions were identified. Since genes that contained regions complementary to the 3′ pairing region, such as *RPL28* and *MYO5B*, were shown to be unaffected by the *RMRP*-derived endo-siRNA (Figure 3B), we searched for candidates based on complementarity of the seed region only. Four open access algorithms, namely TargetScan, microRNA.org, DIANA and TargetRank [14], were used to predict target genes. The most promising candidate target of the endo-siRNA was the gene encoding phytanoyl-CoA 2-hydroxylase interacting protein-like (*PHYHIPL*); this gene was ranked as the highest (DIANA [15] and TargetRank [16]), second highest (TargetScan [17]) or seventh highest (microRNA.org [18]) predicted target score (Supplementary Tables S1–S4). The 3′ UTR of *PHYHIPL* is 2182 nucleotides in length and contains three possible endo-siRNA target sites (Figure 4B). All three sites are 7-mers, which are reported to be more effective than 6-mers [12,13]. In addition, two of the sites are evolutionally conserved.

To investigate whether *PHYHIPL* is a real target of the endo-siRNA derived from *RMRP*, HeLa and 293T cells were transfected with sequence-specific inhibitors of the endo-siRNA, and expression levels of *PHYHIPL* mRNA were analyzed by RT-PCR. Sequence-specific inhibition of the *RMRP*-derived endo-siRNA increased the endogenous expression levels of *PHYHIPL* mRNA in both cell types (Figure 4C). HeLa and MCF7 cells were then transfected with luciferase reporter plasmids containing the wild-type or various deletion mutants of the *PHYHIPL* 3′ UTR. Compared with control cells, luciferase activity was significantly decreased in cells expressing the wild-type *PHYHIPL* 3′ UTR; however, this suppressive effect was attenuated by deletion of each or all of the three endo-siRNA target sequences (Figure 4D). Taken together, these data indicate that the endo-siRNA derived from *RMRP* negatively regulates *PHYHIPL*, and this regulation involves sequences complementary to the seed region.

## 3. Experimental Section

### 3.1. Cell Culture

HeLa, 293T and MCF7 cells were cultured in DMEM containing 10% heat-inactivated FBS at 37 °C in a humidified CO_2_ incubator.

### 3.2. Plasmids

Luciferase reporter vectors (Figure 2) were constructed by cloning a binding site complementary (RMRP-AS 43-22) or identical (RMRP 22-43) to the endo-siRNA sequence into the *Xho*I and *Not*I sites of the psiCHECK-2 vector (Promega, Maddison, WI, USA). The sequences of the binding sites were 5′-AGTCCTCAGTGTGTAGCCTAGG-3′ (sense) and 5′-CCTAGGCTACACACTGAGGACT-3′ (antisense) for RMRP-AS 43-22 and 5′-CCTAGGCTACACACTGAGGACT-3′ (sense) and 5′-AGTCCTCAGTGTGTAGCCTAGG-3′ (antisense) for RMRP 22-43. The vectors containing mutations in the sequence complementary to the endo-siRNA (Figure 4A) were constructed by cloning the following sequences into the *Xho*I and *Not*I sites of psiCHECK-2 Vector: 5′-AGTCCTCAGTGTGTCTAAGCTG-3′ (sense) and 5′-CAGCTTAGACACACTGAGGACT-3′ (antisense) for the seed mutation and 5′-AGTCCTCCTGCTGTCTAAGCTG-3′ (sense) and 5′-CAGCTTAGACAGCAGGAGGACT-3′ (antisense) for the seed/12–15 mutation.

The 3′ UTR of *PHYHIPL* was PCR amplified from HeLa genomic DNA using KOD Plus DNA polymerase (TOYOBO, Osaka, Japan) and then cloned into the *Xho*I and *Not*I sites of psiCHECK-2 Vector. The seed-pairing sequences in the 3′ UTR of *PHYHIPL*, positioned at nucleotides 244–249, 441–447 and 1459–1501, were deleted from the vector using QuikChange XL Site-Directed Mutagenesis Kit (Stratagene, La Jolla, CA, USA). All of the plasmids were verified by DNA sequencing.

### 3.3. Immunoprecipitation of Human AGO2 Complexes

HeLa cells were lysed in lysis buffer A (20 mM Tris-HCl, pH 7.4, 150 mM NaCl, 0.5% NP-40, 0.1 mM dithiothreitol) and immunoprecipitation was performed using Protein G Agarose (Roche Applied Science, Mannheim, Germany) with mouse normal IgG (SC-2025, Santa Cruz Biotechnology, Santa Cruz, CA, USA) or Anti Human Ago2, Monoclonal Antibody (015-22031, Wako Pure Chemical Industries, Osaka, Japan). RNA was isolated from the protein G beads using a miRNeasy Mini Kit (QIAGEN, Hilden, Germany). A portion of the Protein G beads was boiled with 2X loading buffer (0.1 M Tris-HCl, pH 6.8, 4% SDS, 20% Glycerol, 2% β-mercaptoethanol). The supernatants were separated on a SDS-polyacrylamide gel and transferred to a PVDF membrane. The membrane was blocked, incubated with the anti-human AGO2 antibody followed by a horseradish peroxidase-linked secondary antibody (NA931V, GE Healthcare, Buckinghamshire, UK) and then subjected to enhanced chemiluminescence.

### 3.4. Sequence-Specific Inhibition of the *RMRP*-Derived Endo-siRNA

miScript Inhibitor Negative Control was purchased from QIAGEN. miScript miRNA Inhibitor specific for the *RMRP*-derived endo-siRNA was designed and manufactured by QIAGEN. For RT-PCR (Figures 3B and 4C), cells were cultured in 6-well dishes and transfected with 250 pmol of the miScript Inhibitors using Lipofectamine 2000 Reagent (Invitrogen, Carlsbad, CA, USA), according to the manufacturer’s protocol. Cells were harvested 48 h post-transfection using TRIzol reagent (Invitrogen, Carlsbad, CA, USA). For measurements of luciferase activity (Figure 2B), cells were co-transfected with the psiCHECK-2 vectors and 50 pmol of the miScript Inhibitors.

### 3.5. RT-PCR and qRT–PCR

Total RNAs were extracted using RNeasy Mini Kit (QIAGEN, Hilden, Germany). The following primers were used: *RMRP* (5′-TGCTGAAGGCCTGTATCCT-3′ and 5′-TGAGAATGAGCCCCGTGT-3′), *NICN1* (5′-CATCACCACTGTGGCTGTC-3′ and 5′-CTCTGTCAGTGCCCACATC-3′), *MYO5B* (5′-TACAGCCAGTGCACAAGGGTCTGG-3′ and 5′-AAGAAGGGCAGCTGGTTGCGT-3′), *PHYHIPL* (5′-CGCGCCTGGATCATGCCCTC-3′ and 5′-TGCGTCCTGCAATCACTTCAGCC-3′) and *GAPDH* (5′-CTCAGACACCATGGGGAAGGTGA-3′ and 5′-ATGATCTTGAGGCTGTTGTCATA-3′). Reverse transcription was performed for 60 min at 42 °C and then PCR was immediately performed. The PCR conditions included 20–35 cycles of 95 °C for 30 s, 60 °C for 30 s and 72 °C for 30 s (20 cycles for *RMRP*, 28 cycles for *GAPDH*, 30 cycles for *MYO5B* and *PHYHIPL* and 35 cycles for *NICN1*). Signal intensity of PCR products was measured using ImageJ software (version 1.40g; NIH: Bethesda, MD, USA).

Quantification of mRNA, the *RMRP*-derived endo-siRNA, hsa-miR-603 and hsa-miR-645 was performed by TaqMan real-time PCR (Applied Biosystems, Foster City, CA, USA), according to the manufacturer’s protocol. The following TaqMan Gene Expression Assays and TaqMan miRNA Assays (Applied Biosystems, Foster City, CA, USA) were used: *RPL28* (Hs00357189_g1), *GAPDH* (Hs99999905_m1), hsa-miR-603 (001566), hsa-miR-645 (001597) and *RNU6B* (001093). *RNU6B* and *GAPDH* were used as references. A custom TaqMan Small RNA Assay targeting nucleotides 22–43 of *RMRP* was designed and manufactured by Applied Biosystems. For qRT-PCR of hsa-miR-1224-5p, Hs_miR-1224-5p_1 miScript Primer Assay (QIAGEN, Hilden, Germany) was used. Real-time PCR was performed in triplicate for all samples using ABI PRISM 7000 Sequence Detection System (Applied Biosystems, Foster City, CA, USA).

### 3.6. Luciferase Assay

Cells were cultured in 24-well dishes and transfected with 50 ng of luciferase reporter plasmids using FuGENE HD Transfection Reagent (Promega, Maddison, WI, USA), according to the manufacturer’s protocol. In experiments using miScript Inhibitors, the plasmids were transfected with Lipofectamine 2000 Reagent (Invitrogen, Carlsbad, CA, USA), according to the manufacturer’s protocol. Luciferase activity was measured 48 h after transfection using Dual-Luciferase Reporter Assay System (Promega, Maddison, WI, USA).

### 3.7. Statistics

Statistical analyses were performed with Statcel3 software (OMS, Saitama, Japan) using unpaired Student’s *t*-tests. *p* < 0.05 was considered significant.

## 4. Conclusions

The gene silencing technique mediated by siRNA is widely accepted in laboratories, and many researchers are now engaged in the development of therapeutic applications of this method. Previous reports described specific silencing of intended genes by siRNAs; however, emerging evidence has revealed that exogenous siRNAs also affect expression of unintended genes via an off-target effect. Although the off-target effect complicates the therapeutic potential of specific gene targeting by exogenous siRNAs, it also suggests possible expansion of known gene regulation by endogenous siRNAs. Here, we demonstrated that the *PHYHIPL* gene is regulated by an endo-siRNA derived from *RMRP*. To our knowledge, this is the first report of off-target effect of human endogenous siRNAs that is generated through RdRP activity. We elucidated that the endo-siRNA regulates off-target transcripts with seed-matched sequences at their 3′ UTR; however, it should be noted that exogenous introduction of synthetic “*RMRP*-derived endo-siRNA” will demonstrate a different off-target profile through inflammatory response and/or saturation of endogenous RNA interfering machinery [6], as reported for synthetic miRNA [19,20].

A previous report described miRNA-like functions of small nucleolar RNA-derived small RNAs in human cells [21]. These small RNAs are not miRNAs or endo-siRNAs, but they are processed by Dicer cleavage, are associated with AGOs and repress the expression of seed-matched targets. We speculate that the RNA interference machinery in human cells may not strictly discriminate between small RNA species with different biogenetic pathways. As demonstrated here and previously [21–23], human endo-siRNAs and similar small RNAs associate with AGO proteins and regulate expression of multiple genes in the same manner as miRNAs.

## Supplementary Information



## Figures and Tables

**Table 1:** Candidate sequences of the endo-siRNA derived from *RMRP*.

Observed sequence	Nucleotide position of *RMRP*		Number of reads

	5′ end	3′ end	
CTGAAGGCCTGTATCCTAGGCT	8	29	1
CCTAGGCTACACACTGAGGACT	22	43	1
CCTAGGCTACACACTGAGGAC	22	42	1
ACTGAGGACTCTGTTCCTCCCC	34	55	1
ACTGAGGACTCTGTTCCTCCC	34	54	1
TGAGGACTCTGTTCCTCCCCTT	36	57	1
