# Supplementary material for: Off-Target Effect of Endogenous siRNA Derived from RMRP in Human Cells
Source: Int J Mol Sci. 2013 Apr 29;14(5):9305–18. doi: 10.3390/ijms14059305 (PMC3676784; doi:10.3390/ijms14059305)
Supplement: Supplementary file 1 [file ijms-14-09305-s001.pdf]

Supplementary Information

Figure S1. Expression levels of the seed-overlapping miRNAs. (**A**) Seed sequences of three human mature miRNAs located within nucleotides 22–43 of the *RMRP*-derived endo-siRNA sequence. **(B**) qRT-PCR analyses of expression levels of the seed-overlapping mature miRNAs in Hela, 293T and MCF7 cells. Data are represented as the mean ± SD of *n* = 3 independent experiments and are normalized to expression of the *RNU6B* gene. ND, not detected.

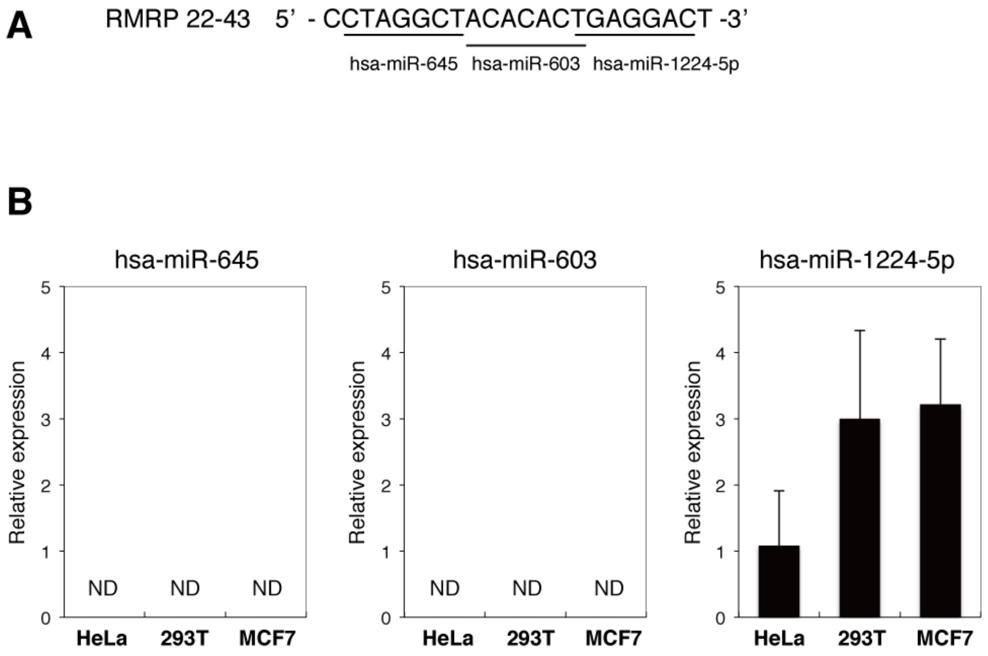

Table S1. Top 20 of the predicted targets TargetScanHuman Release 5.2: June 2011.

Target gene	Gene name	Conserved sites				Poorly conserved sited				Representative miRNA	Total context score	Aggregate PCT
		total	8mer	7mer-m8	7mer-1A	total	8mer	7mer-m8	7mer-1A			
RAB22A	RAB22A, member RAS oncogene family	1	1	0	0	2	1	1	0	hsa-miR-645	−0.78	N/A
PHYHIPL	phytanoyl-CoA 2-hydroxylase interacting protein-like	1	1	0	0	2	0	1	1	hsa-miR-645	−0.73	N/A
B3GALNT1	beta-1,3-N-acetylgalactosaminyltransferase 1 (globoside blood group)	1	1	0	0	1	0	1	0	hsa-miR-645	−0.67	N/A
TNKS	tankyrase, TRF1-interacting ankyrin-related ADP-ribose polymerase	1	1	0	0	1	1	0	0	hsa-miR-645	−0.63	N/A
SOX30	SRY (sex determining region Y)-box 30	1	1	0	0	0	0	0	0	hsa-miR-645	−0.53	N/A
GJC1	gap junction protein, gamma 1, 45kDa	1	0	1	0	3	2	0	1	hsa-miR-645	−0.53	N/A
UBA2	ubiquitin-like modifier activating enzyme 2	1	1	0	0	0	0	0	0	hsa-miR-645	−0.5	N/A
SMARCA1	SWI/SNF related, matrix associated, actin dependent regulator of chromatin, subfamily a, member 1	1	1	0	0	0	0	0	0	hsa-miR-645	−0.5	N/A
APPL2	adaptor protein, phosphotyrosine interaction, PH domain and leucine zipper containing 2	1	1	0	0	0	0	0	0	hsa-miR-645	−0.48	N/A
MEF2A	myocyte enhancer factor 2A	1	1	0	0	0	0	0	0	hsa-miR-645	−0.47	N/A
STK40	serine/threonine kinase 40	1	1	0	0	0	0	0	0	hsa-miR-645	−0.45	N/A
ZFAND3	zinc finger, AN1-type domain 3	1	1	0	0	0	0	0	0	hsa-miR-645	−0.45	N/A
ZNF664	zinc finger protein 664	1	1	0	0	0	0	0	0	hsa-miR-645	−0.42	N/A
SLC8A1	solute carrier family 8 (sodium/calcium exchanger), member 1	1	1	0	0	1	0	0	1	hsa-miR-645	−0.42	N/A
PPM1E	protein phosphatase 1E (PP2C domain containing)	1	1	0	0	0	0	0	0	hsa-miR-645	−0.41	N/A
SPRY3	sprouty homolog 3 (Drosophila)	1	1	0	0	1	0	0	1	hsa-miR-645	−0.41	N/A
WNK1	WNK lysine deficient protein kinase 1	1	1	0	0	0	0	0	0	hsa-miR-645	−0.39	N/A
TBC1D20	TBC1 domain family, member 20	1	1	0	0	1	0	0	1	hsa-miR-645	−0.38	N/A
MGC24039	hypothetical protein MGC24039	1	1	0	0	0	0	0	0	hsa-miR-645	−0.37	N/A
XPO1	exportin 1 (CRM1 homolog, yeast)	1	1	0	0	0	0	0	0	hsa-miR-645	−0.37	N/A

Table S2. Top 20 of the predicted targets microRNA.org-Target and Expression August 2010 Release.

mRNAs	mirSVR score
SPANXN1	−2.89
TMX3	−1.83
RUNDC2C	−1.54
RAB5A	−1.54
B3GALNT1	−1.54
MCL1	−1.53
PHYHIPL	−1.41
SMARCA1	−1.34
GCA	−1.33
SOX30	−1.33
UBA2	−1.32
CREM	−1.32
ZNF521	−1.32
NF1	−1.32
APPL2	−1.3
CCDC66	−1.29
SNORA81	−1.29
DACH1	−1.29
LOC645166	−1.28
ZNF449	−1.28

Table S3. Top 20 of the predicted targets DIANA-microT-CDS v.4.

Ensembl Gene Id	miRNA name	miTG score	SNR	Precision
ENSG00000165443 (PHYHIPL)	hsa-miR-645	0.766	1.3	0.8
ENSG00000198739 (LRRTM3)	hsa-miR-645	0.641	1.3	0.2
ENSG00000068305 (MEF2A)	hsa-miR-645	0.619	1.3	0.2
ENSG00000187605 (TET3)	hsa-miR-645	0.605	1.3	0.2
ENSG00000176887 (SOX11)	hsa-miR-645	0.599	1.3	0.2
ENSG00000134644 (PUM1)	hsa-miR-645	0.593	1.3	0.2
ENSG00000173273 (TNKS)	hsa-miR-645	0.593	1.3	0.2
ENSG00000198795 (ZNF521)	hsa-miR-645	0.566	1.3	0.2
ENSG00000126261 (UBA2)	hsa-miR-645	0.546	1.3	0.2
ENSG00000165659 (DACH1)	hsa-miR-645	0.541	1.3	0.2
ENSG00000183023 (SLC8A1)	hsa-miR-645	0.539	1.3	0.2
ENSG00000168575 (SLC20A2)	hsa-miR-645	0.52	0.9	0.2
ENSG00000217473 (PEG10)	hsa-miR-645	0.52	1	0.2
ENSG00000102038 (SMARCA1)	hsa-miR-645	0.519	1	0.2
ENSG00000106991 (ENG)	hsa-miR-645	0.511	1	0.2
ENSG00000165516 (KLHDC2)	hsa-miR-645	0.504	0.9	0.2
ENSG00000110881 (ACCN2)	hsa-miR-645	0.504	1	0.2
ENSG00000156639 (ZFAND3)	hsa-miR-645	0.503	1	0.2
ENSG00000180304 (OAZ2)	hsa-miR-645	0.502	1	0.2
ENSG00000060237 (WNK1)	hsa-miR-645	0.501	1.1	0.2

Table S4. Top 20 of the predicted targets TargetRank.

Rank	Gene Name	Gene/Isoform Description	TargetRank Score	Seed Match Total	Conserved Seed Matches				Non-conserved Seed Matches			
					8mer	M8 7mer	A1 7mer	6mer	8mer	M8 7mer	A1 7mer	6mer
1	PHYHIPL	phytanoyl-CoA 2-hydroxylase interacting	0.6450	3	1	0	1	0	0	1	0	0
2	RAB22A	RAS-related protein RAB-22A	0.5690	4	0	0	0	0	2	1	0	1
3	RANBP10	RAN binding protein 10	0.5200	4	0	0	0	0	1	2	1	0
4	SLC5A3	solute carrier family 5 (inositol transporters)	0.5180	2	0	0	0	0	1	1	0	0
5	MCFD2	multiple coagulation factor deficiency 2	0.5150	4	0	0	0	0	1	2	0	1
6	TNKS	tankyrase - TRF1-interacting ankyrin-related	0.5100	2	0	0	0	0	2	0	0	0
6	PCDH11X	protocadherin 11 X-linked isoform d	0.5100	2	0	0	0	0	2	0	0	0
6	PCDH11Y	protocadherin 11 Y-linked isoform c	0.5100	2	0	0	0	0	2	0	0	0
9	FSTL4	follistatin-like 4	0.5090	4	0	0	0	0	1	1	2	0
10	GJA7	connexin 45	0.5050	4	0	0	0	0	2	1	1	0
11	SLC8A1	solute carrier family 8	0.4900	3	0	0	1	0	1	0	0	1
12	ACVR1B	activin A type IB receptor isoform a precursor	0.4540	2	0	0	0	0	2	0	0	0
13	KLHL12	kelch-like 12	0.4380	2	0	0	0	0	1	1	0	0
14	WDR32	WD repeat domain 32	0.4340	4	0	0	0	0	0	4	0	0
15	LPAL2	lipoprotein - Lp(a)-like 2 precursor	0.4130	2	0	0	0	0	2	0	0	0
16	PIP3-E	phosphoinositide-binding protein PIP3-E	0.4060	3	0	0	0	0	1	1	1	0
17	KCNN1	potassium intermediate/small conductance	0.4000	4	0	0	0	0	2	0	0	2
18	MCL1	myeloid cell leukemia sequence 1 isoform 1	0.3960	2	0	0	0	0	0	2	0	0
18	MCL1	myeloid cell leukemia sequence 1 isoform 2	0.3960	2	0	0	0	0	0	2	0	0
20	LIMD1	LIM domains containing 1	0.3940	2	0	0	0	0	0	0	2	0
20	MGC22014	hypothetical protein LOC200424	0.3940	2	0	0	2	0	0	0	0	0

© 2013 by the authors; licensee MDPI, Basel, Switzerland. This article is an open access article distributed under the terms and conditions of the Creative Commons Attribution license (http://creativecommons.org/licenses/by/3.0/).
